# Sound Source Localization through 8 MEMS Microphones Array Using a Sand-Scorpion-Inspired Spiking Neural Network

**DOI:** 10.3389/fnins.2016.00479

**Published:** 2016-10-27

**Authors:** Christoph Beck, Guillaume Garreau, Julius Georgiou

**Affiliations:** Holistic Electronics Research Lab, Department of Electrical and Computer Engineering, University of CyprusNicosia, Cyprus

**Keywords:** bio-inspired, acoustic localization, neuromorphic, spiking-neurons, microphone array

## Abstract

Sand-scorpions and many other arachnids perceive their environment by using their feet to sense ground waves. They are able to determine amplitudes the size of an atom and locate the acoustic stimuli with an accuracy of within 13° based on their neuronal anatomy. We present here a prototype sound source localization system, inspired from this impressive performance. The system presented utilizes custom-built hardware with eight MEMS microphones, one for each foot, to acquire the acoustic scene, and a spiking neural model to localize the sound source. The current implementation shows smaller localization error than those observed in nature.

## Introduction

Scorpions belong to the most ancient groups of terrestrial animals. Based on the findings of preserved fossils it is believed that the order scorpion is more than 430 million years old. Most scorpions are nocturnal and seek shelter in the day for protection from predators and predominately live in sandy or rocky subsoil close to the ground in the tropics, subtropics, semi-deserts, or deserts.

Their anatomy basically consists of the anterior part of the body (Prosoma), posterior part of the body (Mesosoma), tail with sting (Metasoma), eight legs, claws (Pedipalbs), and pincers (Chelae).

Some species like the sand scorpion, e.g., Paruroctonus Mesaensis, perceive their environment via a unique and fascinating method—they sense ground waves with their basitarsal compound slit sensilla (BCSS), located just above the joint of the foot (Tarsus; Stürzl et al., 2000). Due to this anatomy and the very highly sensitive organs, the scorpions can detect ground waves of < 0.1 nm amplitude, which is around the size of an atom.

By measuring the time delay between the waves arriving at each of its feet, the scorpion can calculate the precise direction and distance to a threat, a predator, a prospective prey, or even a mate.

The ability to perceive the environment based on vibrations is something we humans share with scorpions. Whilst they sense ground waves, we detect vibrations in the air—sound. Based on this link we propose a system that adapts a sand scorpion inspired neuronal model to human audible sound.

### Related work

Prior work for bioinspired acoustic surveillance units (ASU; from flies), such as that of Cauwenberghs et al. used spatial and temporal derivatives of the field over a sensor array of MEMS microphones, power series expansion, and Independent Component Analysis (ICA) for localizing and separating mixtures of delayed sources of sound (Cauwenberghs et al., 2001). This work showed that the number of sources that can be extracted depends strongly on the number of resolvable terms in the series.

Similar work was also done by Sawada et al. using ICA for estimating the number of sound sources (Sawada et al., 2005a) and localization of multiple sources of sound (Sawada et al., 2003, 2005b).

Julian et al. compared four different algorithms for sound localization using MEMS microphones and signals recorded in a natural environment (Julian et al., 2004). The spatial-gradient algorithm (SGA) showed the best accuracy results. The implementation requires a sampled data analog architecture able to solve adaptively a standard least means-square (LMS) problem. The performance of the system, with low power CMOS VLSI design, is of the order of 1° error margin and similar standard deviation for the bearing angle estimation (Cauwenberghs et al., 2005; Julian et al., 2006; Pirchio et al., 2006). A very low power implementation for interaural time delay (ITD) estimation without delay lines with the same ASU unit is reported by Chacon-Rodriguez et al. with an estimation error in the low single-digit range (Chacon-Rodriguez et al., 2009).

Masson et al. used a data fusion algorithm to calculate the estimation of the position based on measurements from five nodes, each with four MEMS microphones (Masson et al., 2005). The measurement is made from a unique fixed source emitting a 1 kHz signal.

Zhang and Andreou used cross correlation of the signals received and a zero crossing point to estimate the bearing angle of a moving vehicle (Zhang and Andreou, 2008). The hardware was an ASU with four MEMS microphones.

### Neuronal networks

The highlighted algorithms have demonstrated good results but seem computationally hungry in comparison to using a neural network approach. The advantages of neuronal networks compared to other digital algorithm are discussed in Orponen (1994) and Goutte (1996).

No consensual theory of ITD-based localization existed until Benichoux et al. recently proposed a unifying model of the function of binaural cells in mammals that explains the behavioral and neural data alike (Benichoux et al., 2013).

However, neuromorphic implementations have already been proposed, such as a neuromorphic sound localizer reported by (van Schaik and Shamma, 2003, 2004). Here, two MEMS microphones are connected to two analog neuromorphic cochlea. This system has low power consumption and the average standard deviation of the estimated azimuth is 4.5° (van Schaik and Shamma, 2003, 2004). This system bases its azimuth estimation on the interaural delay between the two “ears.”

In our previous work (Garreau et al., 2013), we have demonstrated a system that performs person localization based on ground waves fed to a bio-inspired spiking neuron model without using ITD. The vibration data was collected using five seismic sensors (Mark Product L-15B 4.5 hz x,y,z geophones) positioned at the center of a circle. The model improves upon the model of sand-scorpions of Stürzl et al. (Stürzl et al., 2000).

## Materials and methods

### System

In this work, we extend the above-mentioned methods, inspired by the nocturnal sand scorpion, to waves traveling in air. The aim is to create a surveillance unit that can be used in localizing a speaker during a conversation, conference, or videoconference.

One of the features the new generation of videoconference system offers is the automatic zoom of the camera onto the current speaker. For simple systems, when a person wants to speak, he switches his microphone to the position “ON” and the camera turns and zooms to a predefine setting (S. Corporation, 2001; M. T. ltd.^1^). More complex systems have higher flexibility and automatically zoom to the speaker using facial recognition but the location still depends on pre-set places (Polycom^2^; M. T. ltd.^3^). The system presented can localize solely based on the neuronal model and would neither require any pre-sets nor fixed or static locations of the speakers.

**Figure 1:**
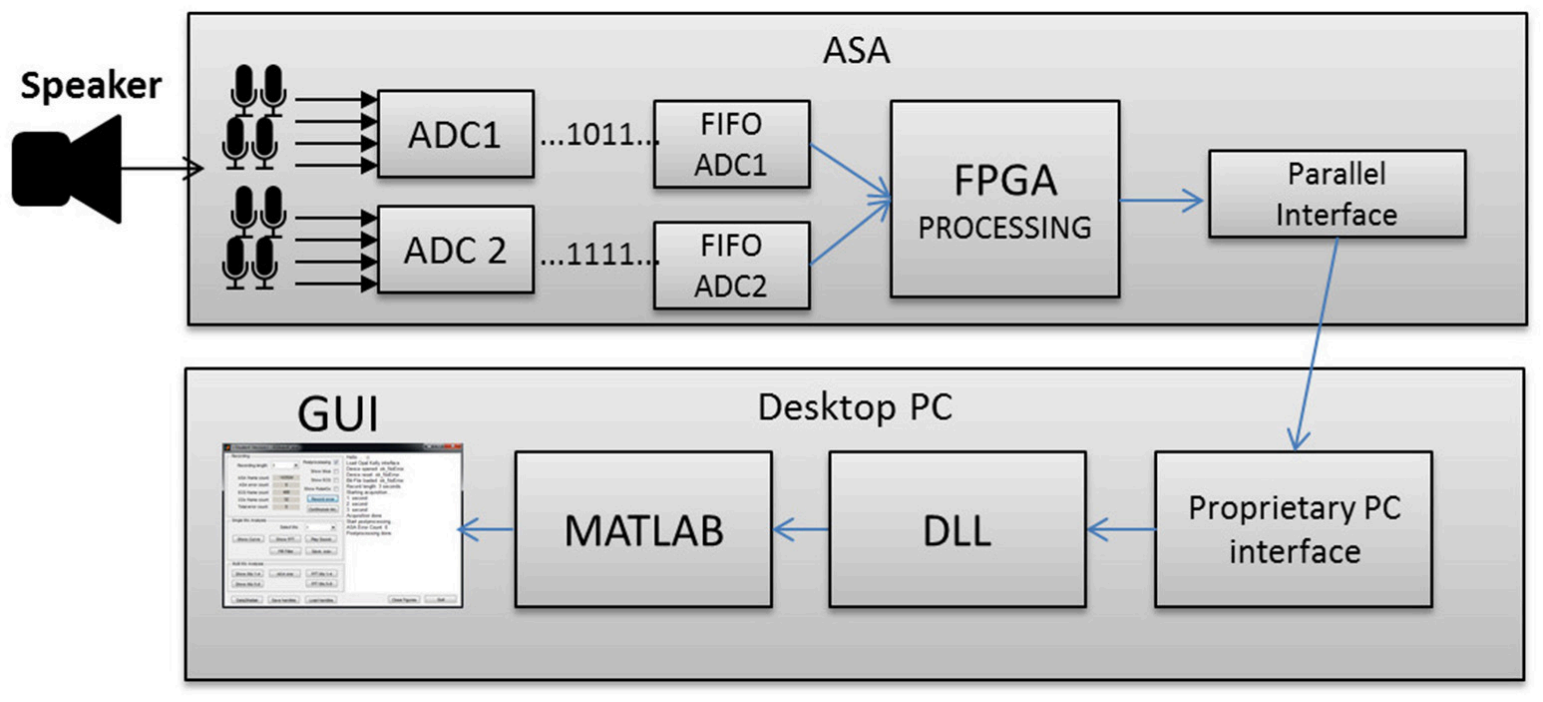
**System block diagram**.

In this work we propose a system consisting of a custom built acoustic sensor array (ASA) with a circular array of eight MEMS microphones, a third-party board interfacing the ASA to a PC, a custom designed MATLAB® application to control the ASA and an adapted bioinspired neuronal sound source localization model. The system diagram is shown in Figure 1.

#### Acquisition hardware

**Figure 2:**
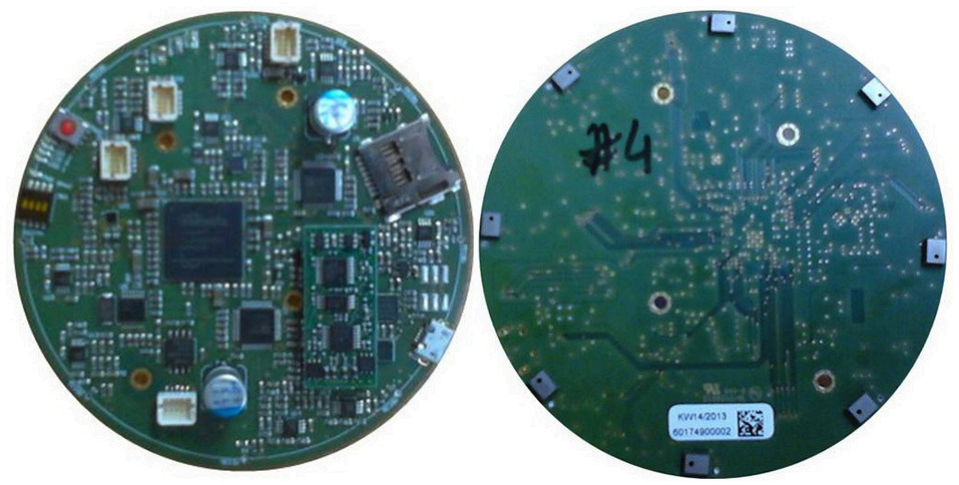
**Picture of the ASA board top (left) and bottom (right)**.

**Figure 3:**
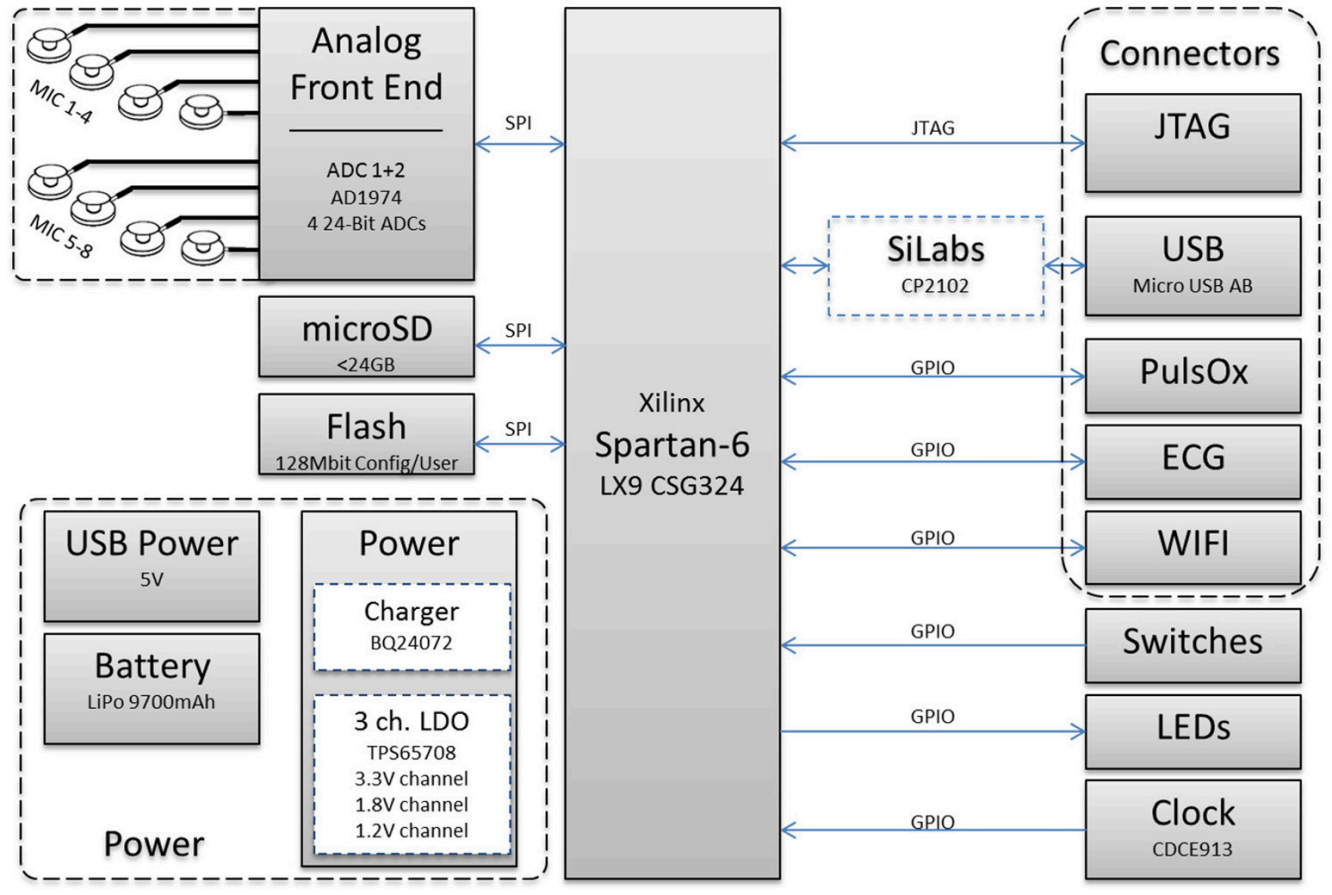
**Hardware block diagram**.

The custom designed hardware ASA is a battery powered circular four layers PCB with a diameter of 90 mm as shown in Figure 2, the hardware block diagram is shown in Figure 3, respectively. The PCB holds the required circuits on the front side (left), and eight equally spaced, omnidirectional Knowles® SPM0408HE5H MEMS microphones, placed on the outside edge, perpendicular to the PCB on the back side (right). The microphones are pre-amplified and have a sensitivity of –22 dB SPL at 1 kHz. The current consumption of each microphone is < 200 μA and the form factor is 4.7 × 3.7 mm. The system is intended to record within the human audible frequency range plus a solid safety margin, resulting in 24 kHz, the Nyquist frequency of 48 kHz. Close to each microphone is a two stage differential amplifier that includes basic band-pass filtering which gives a flat response between 1.6 Hz and 24 kHz. These amplifiers increase the input signal and differentially forward the signals to two four-channel Analog Devices AD1974 ADCs (not multiplexed) with multi-bit sigma-delta conversion architecture at 24-bits resolution. The architecture guarantees a good noise shape for lower frequency applications, such as speech. Both ADCs are sourced by the same clock to ensure low jitter. This is especially important for time correlated signals and the time of arrival based algorithm.

Core to the mobile unit is a powerful Xilinx Spartan-6® LX9 CSG324 FPGA. It was chosen with a generous safety margin related to computing power, speed and pin count at the expense of power consumption, implementation space, and complexity. This provides real-time onboard processing capabilities for all eight acoustic channels. The FPGA is connected to the ADCs via independent SPI interfaces and provides a parallel 17-bit raw data interface with a bandwidth of ~11 Mbit/s. The system is either USB-powered or supplied from a lithium polymer battery. The battery can maintain the mobile unit running for ~5 h.

#### Embedded application

Firstly the embedded application receives the data from the ADCs and adds meta-information such as channel number, time, and CRC. It is important to maintain the time correlation of all channels because the sand scorpion neuronal model is highly dependent on it. Additionally, measures to ensure data integrity were developed that followed well-known communication methodologies, including framing and error detection.

**Figure 4:**
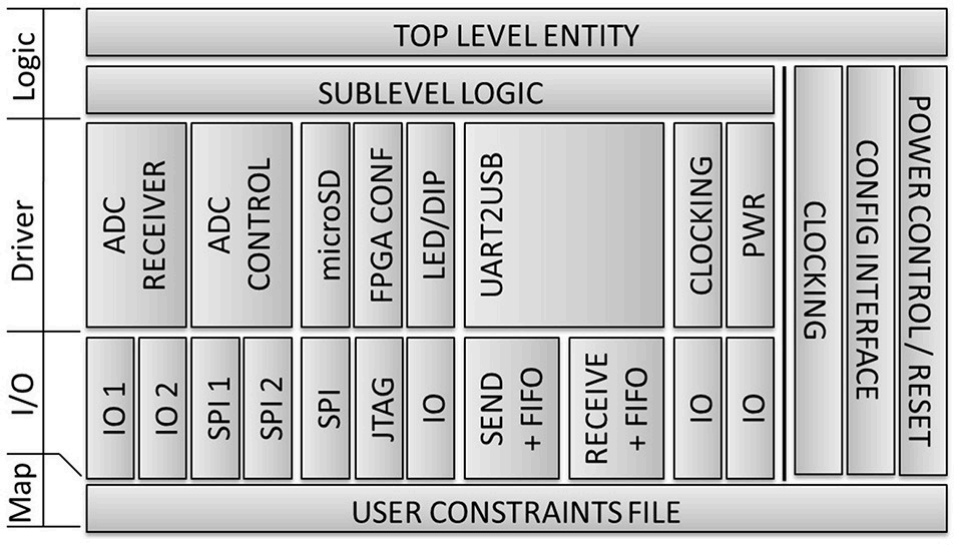
**Block diagram of embedded application**.

The FPGA's logic is described using VHDL and the Xilinx ISE® development environment. As shown in the block diagram in Figure 4, it is based on a modular architecture with vertical and horizontal separation. Well-defined module interconnections and a strong focus on well-known design patterns accelerated development, reduced complexity, and increased reliability of the entire system. Various simulation possibilities from Xilinx®, accompanied by a proprietary 17-bit debug interface offered verification facilities during design and on-board testing. The recent implementation comprises 6000+ lines of code using ~38% of the resources available, thus having significant capabilities available for future onboard real-time algorithms.

#### Host application

**Figure 5:**
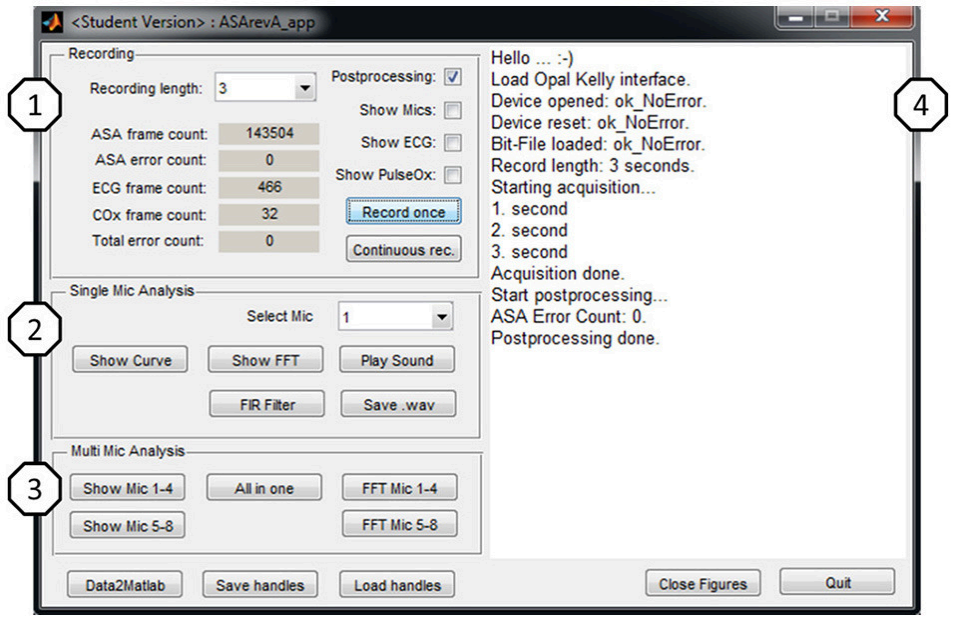
**GUI of MATLAB^®^ application**.

For design and characterization tasks a MATLAB® based host application with Graphical User Interface (GUI) was developed as illustrated in Figure 5. The application provides two modes: a real-time mode to acquire data from the MCU; and an offline mode where stored data-sets can be loaded into the application. This allows algorithm design with MATLAB® and test/comparison against dedicated data-sets.

The GUI is divided into four parts to provide easy access to frequently used functions and convenience features. **Recording (1):** the user can chose a time, select the settings and start a single or continuous recording. The MCU will immediately start recording as configured and transmit the data. **Single Mic Analysis (2):** Some basic data analysis algorithms that focus on one acoustic channel are easily accessible from the GUI to ease handling. More comprehensive analysis is still possible via the MATLAB® command line. **Multi Mic Analysis (3):** Some basic data analysis algorithms that focus on comparison of acoustic channels, e.g., amplitude comparison or time of arrival figures are also accessible from the GUI to accelerate handling, such as standard checks that were often executed. **Log (4):** A text log representing the application's status during runtime. This helps to keep track when working with time scheduled recordings.

**Figure 6:**
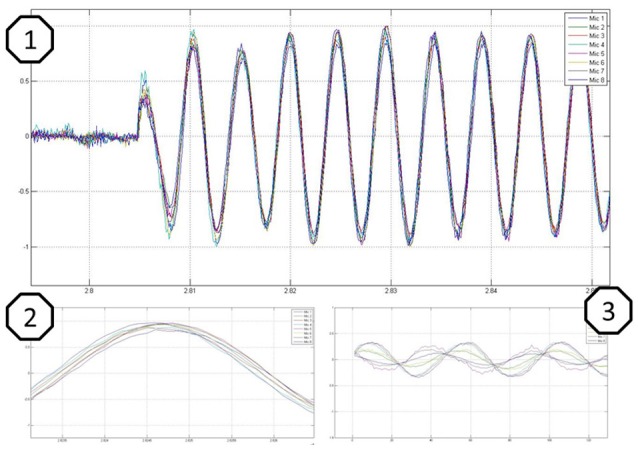
**Sine wave recordings illustrated in diagrams amplitude over time, where 1 shows the switch on behavior, 2 a recording with equal microphones to speaker distance, and 3 a recording with varying microphones to speaker distance**.

When performing predefined single or multi-mic analysis, dedicated figures open to show the recorded data in diagrams. Convenience features are shown in Figure 6. Figure 6.1 shows time synchronized sine waves from all eight channels (samples over time) representing the switch-on behavior, Figure 6.2 shows a single source with equal microphone to speaker distance, and Figure 6.3 illustrates varying microphone to speaker distance.

#### Test set-up

**Figure 7:**
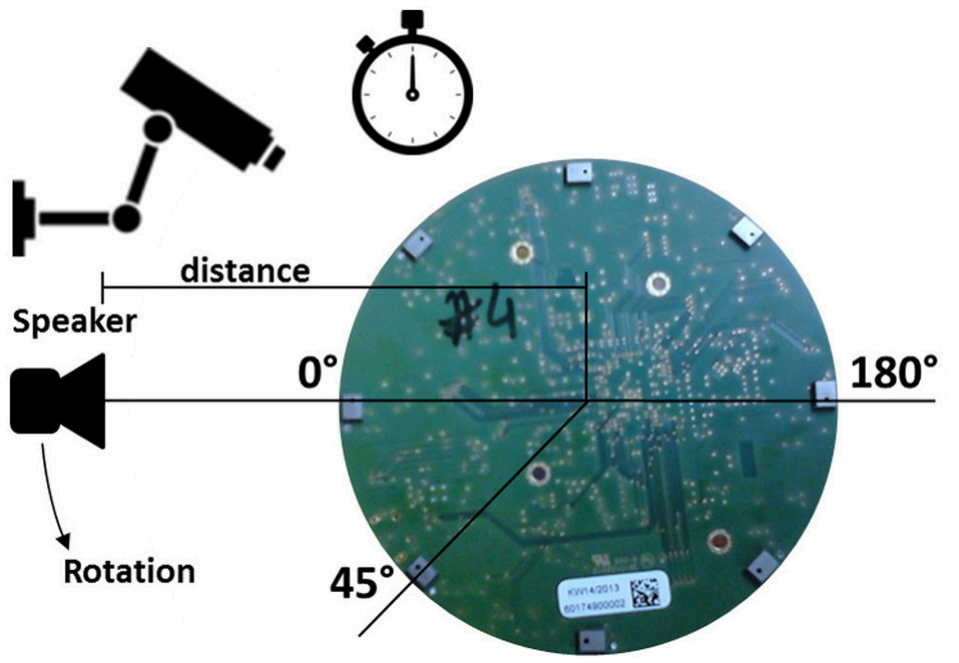
**Schematic setup with video and time surveillance and the speaker placed in the same horizontal plane as the microphones rotating counterclockwise**.

The schematic test set-up is shown in Figure 7. For data collection, the ASA was placed on top of a turntable with the microphones facing upwards. A Gallo Acoustics A'Diva omni-directional speaker was used as the sound source to play back various types of sounds (detailed in next Sub-section). The speaker was placed and rotated in the same horizontal plane (height) as the microphones in order to have a two dimensional set-up. The distance between the speaker and the ASA center point was 1 m and kept constant. The speaker was fixed, while the ASA was rotated at a constant speed from 0° to 360° over a period of 20 s.

The time was recorded to have a reference and a camera continuously recorded the movement from an eagle's eye perspective in order to have a reference deflection angle.

#### Measurements

In total, 20 recordings were collected and analyzed so as to be able to characterize the neural network based localization system and to compare the system accuracy with that found in nature based on measurements taken from real scorpions (Brownell and Farley, 1979).

To investigate the simplest scenario first, 15 time a continuous 1 kHz sine wave in continuous and stepwise (0°, 90°, 180°, and 270°) movement around the ASA were recorded. Then, recordings of sounds reflecting more realistic scenarios were collected: continuous and discontinuous reading and counting and frequency sweeps.

The recent set-up has a power consumption of 350 mW during normal recording. Yet, the system consumes the equivalent to 100% duty cycle, because no power saving mechanisms are implemented.

#### Data processing

The data recorded by our ASA required post-processing steps before being used with the model described above. First, we calculated the maximum, mean, and standard deviation of the signal for each sensor. Second, the mean is removed and the signal is divided by its standard deviation. Third, the resulting signal was normalized to ±1 (divided by its max). This was done in order to remove offsets and gain mismatches that exist between the sensory inputs. That may be attributed to the fact that the microphones are from different batches and the signal path gains vary, as the discrete components have varying tolerances. Finally, for each leg a text file was generated according to the input format definition of the model. Processed data was used as input to the network and neuron activity was monitored over a predefined time window while the model generated a new bearing angle estimation. The processing was done with MATLAB® 2012. The scorpion inspired model was used in the Brian spiking neuron network (SNN) simulator (Goodman and Brette, 2008; Brette and Goldberg, 2009) with Spyder2® environment running on Python2.7®.

### Spiking neural model

The sound source bearing angle was calculated using a spiking neural model. The model was originally developed by biologists who investigated the theory behind sand scorpion prey localization. They have shown that time-coding through spiking neurons is key to this process. First an overview of the original model will be presented and then the modifications required in order to work with the 8 microphone data acquisition board (ASA). Note that in this scenario, no learning is done, the SNN processes the incoming data but doesn't have any memory of past events.

#### Original neural model

**Figure 8:**
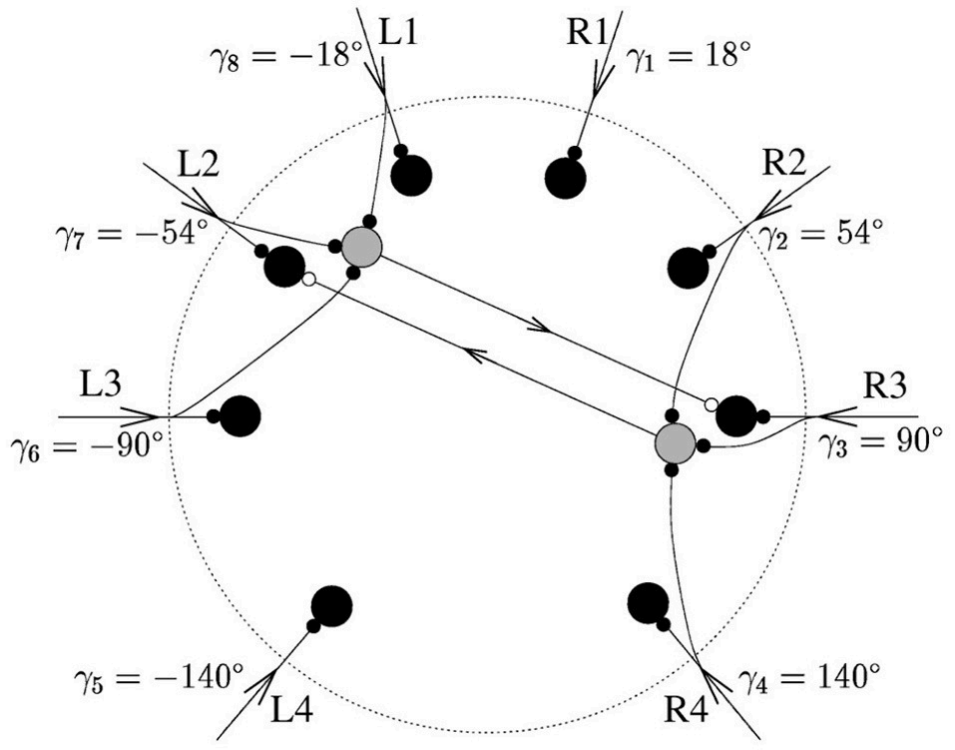
**Original spiking neuron model developed for the sand-scorpion, from Stürzl et al. (2000)** The eight command neurons are colored in black. For two of them, *k* = 3 and *k* = 7 = 3 corresponding to R3 and L2, respectively, the inhibitory partner neurons are shown as well in gray. The triad of R3 consists of L1, L2, and L3.

The model created by Stürzl et al. (2000) as shown in Figure 8 has eight command neurons (black) and eight inhibitory neurons (gray; only two are illustrated). Each one of the eight legs of the arachnid is connected to one command neuron and three opposite inhibitory partner neurons. For example the excitatory command neuron at R3 is connected to the three inhibitory partner neurons at L1, L2, and L3.

The sense organ of each leg of the arachnid, the BCSS, located just above the joint of the foot is compressed as a transverse wave passes along. The reaction mechanism creates an excitatory spike at the command neuron corresponding to this leg, e.g., R3. It also creates an excitatory spike at the three inhibitory partner neurons connected to the leg and projecting their inhibitory spike to the command neurons of the opposite legs, e.g., L1, L2, and L3. After the neuron spike rates, defined as *a*_*j*_, are obtained, the direction of the source must be computed.

Equation (1) shows the formula which calculates *x*, the polar position of the prey, where θ_*j*_ is the angle of the leg *j* and *a*_*j*_ the spike rate over a predefined time window. Equation (2) is used to calculate the computed source angle φ through the arg() function:

(1) $$\begin{array}{rcl} x & = & \;\overset{5}{\underset{j=1}{\sum }}\left(a_{j}\;\cdot \;\exp\left(i\theta_{j}\right)\right) \end{array}$$

(2) $$\begin{array}{rcl} \varphi \; & = & \;arg\left(x\right) \end{array}$$

This model has been implemented on a spiking neural simulator Brian (Goodman and Brette, 2008; Brette and Goldberg, 2009) but the implementation has limitations; the main one being that it uses a single simulated Rayleigh wave that is fed to each leg with a certain delay, which depends on the location of source of the wave and the leg position. The same method was applied when noise was added to the simulated wave, thus exposed each leg to the same (but delayed) noise. This is different to the case of real data, where each sensor/leg has a different characteristic response and completely different noise contributions. Finally, the above model was not tested on moving sources.

#### Modifications of the brette implementation

In order to adapt the Brette implementation to our dataset, two factors needed to be changed. The first one is related to the input signals. The Brette implementation generates one simulated Rayleigh wave and adds delays, calculated according to source location, for the different legs. The modified model is rewritten so as to accommodate the eight unique input signals collected by each of MEMS microphones located on our acquisition system. The second one is about the source characteristics. The code is designed for a fixed location source, whereas we modified it so it can respond to a moving source, i.e., we added a loop that run the simulation with a different set of data at each loop. On the other hand, some features were kept as in the Brette implementation: (a) the refractory index, we let it equal to the max inter-leg delay; (b) the inter-neurons delay was kept at 70% of the max inter leg delay; (c) the excitatory and inhibitory weights were kept at 7 and −2 respectively. The main justification for the changes is to remove the limitations of the Brette implementation.

## Results

By feeding a modified spiking neural network scorpion model with normalized raw data, a new angle value is generated every 500 ms. It is possible to identify and compare the bearing angle of the sound source. In nature, the scorpion has a 13° error on the localization of acoustic stimuli, which when it comes to catching food can be compensated by the size of the pedipalps (Brownell and Farley, 1979).

**Figure 9:**
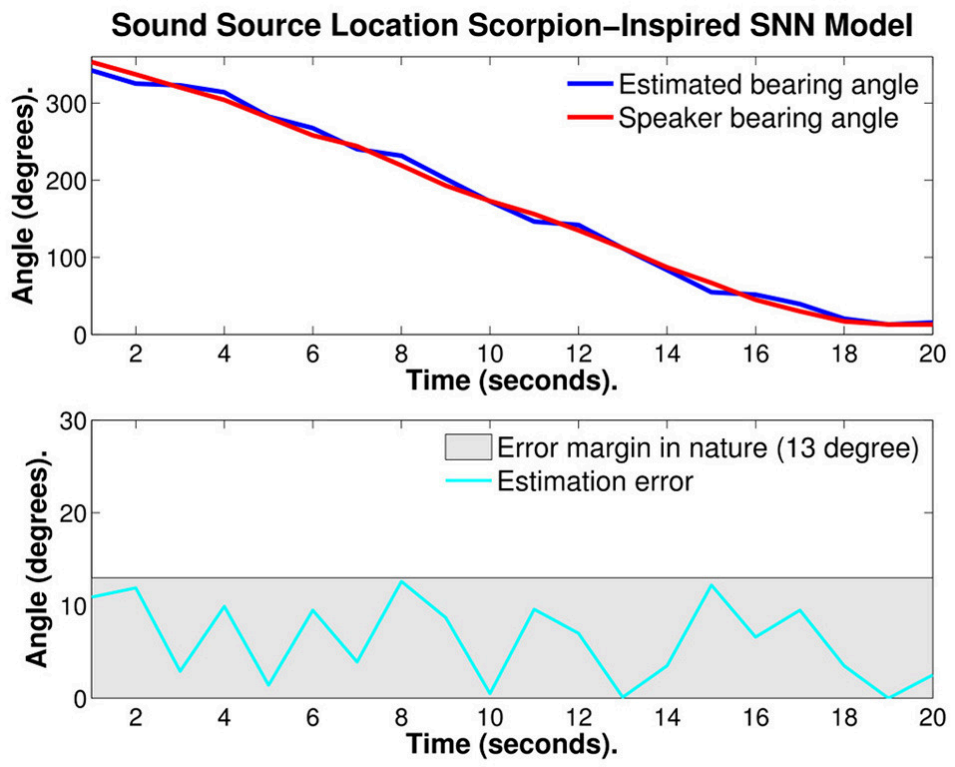
**Plots of: the speaker bearing angle (red), the estimated bearing angle (blue), and the estimation error (cyan)**. The average error reported is 6.34° ± 4.36°.

Figure 9 illustrates the first test, a continuously moving single frequency source. In the top part of the figure, the red curve is the bearing angle of the source of sound (speaker) and the blue curve is the estimated bearing angle of the source calculated with the SNN model. In the bottom of the figure, the estimation error is given. On this example the average estimation error is 6.34° ± 4.36°.

The same example was then recalculated with several refractory periods: 5, 10, 15, and 20%. The 15% value was the one that performed best.

**Figure 10:**
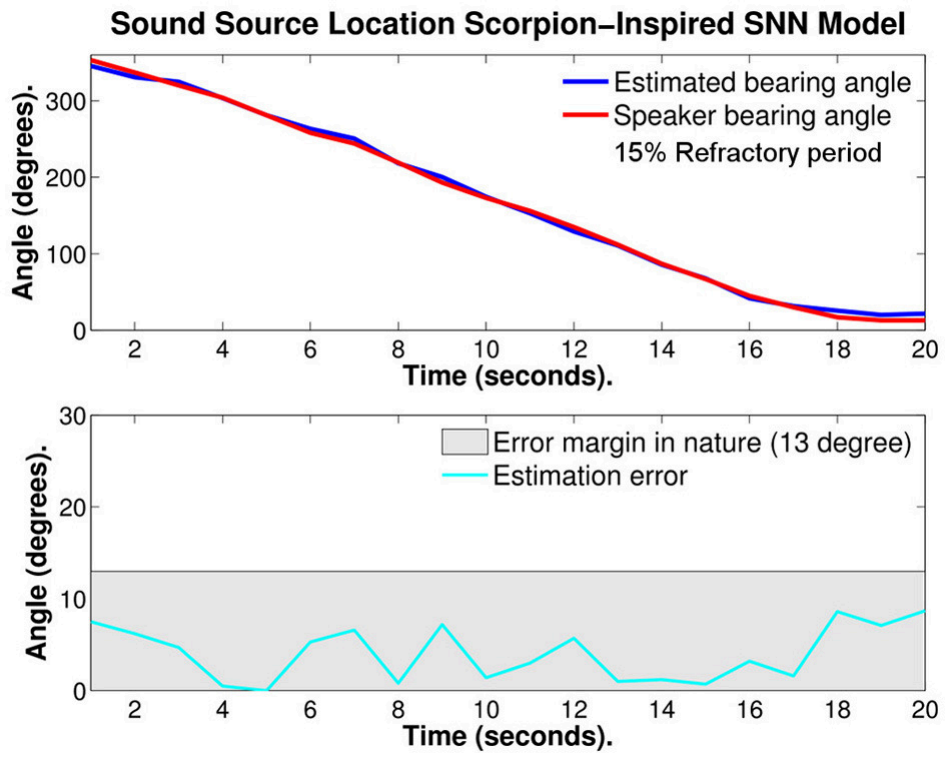
**Plots of: the speaker bearing angle (red), the estimated bearing angle (blue), and the estimation error (cyan) with 15% refractory period**. The average error reported is 4.05° ± 3.01°.

Figure 10 illustrates the same test with 15% refractory period, the red curve is the bearing angle of the source of sound (speaker) and the blue curve is the estimated bearing angle of the source calculated with the SNN model. In the bottom of Figure 10, the estimation error is given. With optimized refractory period the average estimation error was reduced to 4.05° ± 3.01°. This is almost 1/4 of the error observed in nature.

If all 15 trials based on the continuous sine wave scenario were taken into account the average estimation error is 9.6° ± 7.6°.

The major improvement with the refractory period could be explained by the fact that the estimation of the angle is calculated during a window of time during which the source of sound is moving and thus the estimation is an average of the location during that window of time. Adding a refractory period stops the neuron activity and it is similar to resetting the angle estimate and gives more weight to the more recent direction of the source of sound. A too long refractory period underperforms as there isn't enough spike activity for the estimation of the bearing angle.

**Figure 11:**
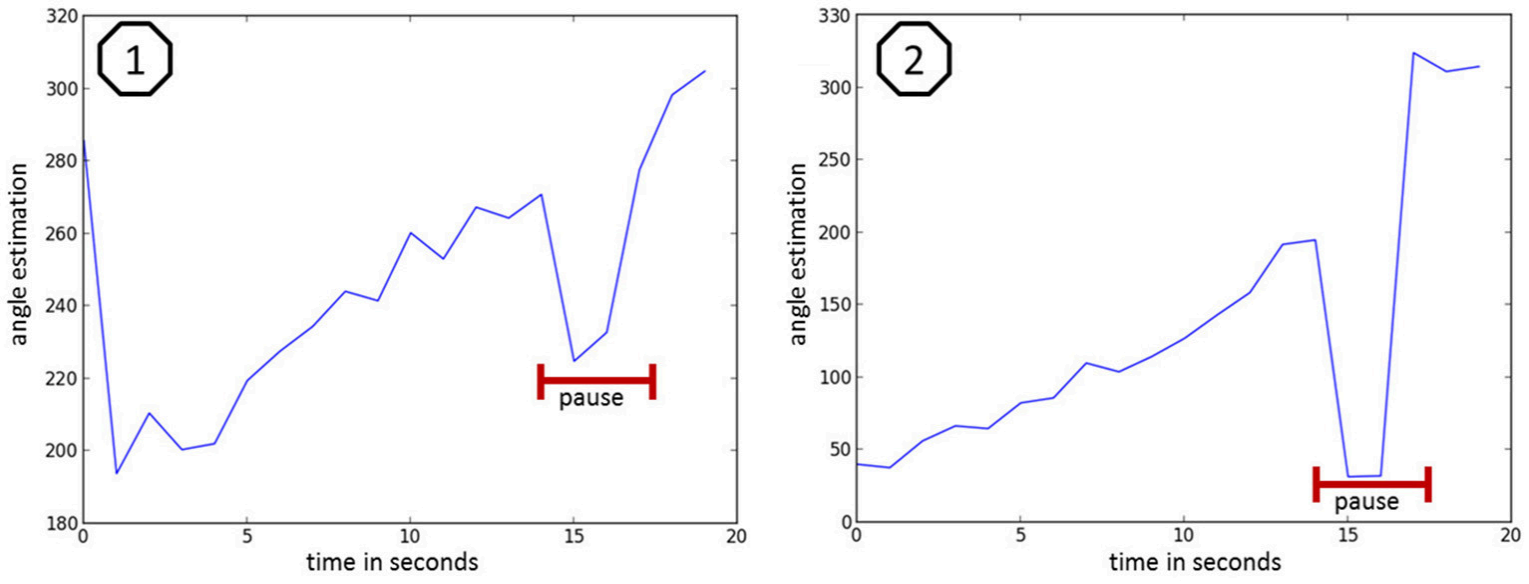
**speaker bearing angle estimation at the example of discontinuous counting, where 1 shows the estimation of someone normally counting (very short breaks within the numbers) with a one second pause at second 16 (marked red) and 2 shows the estimation of someone monotonously counting with a one second pause at second 16 (marked red)**.

Figure 11 illustrates the trajectory estimation at the example of discontinuous counting. Figure 11.1 shows the estimation for someone normally counting (very short breaks within the numbers) with a 1 s pause at second 16 (marked red). Figure 11.2 shows the estimation for someone monotonously counting with a 1 s pause at second 16 (marked red). With the recent setup it was not possible to reliably calculate the position for the speaking person and thus an estimation of the mean error for the bearing angle values was not possible.

A more precise evaluation and improvement of the performance of the model with discontinuous stimuli is under active investigation.

## Discussion

We have demonstrated a system that performs sound source localization through eight circularly placed MEMS microphones on a custom built PCB and a bioinspired, scorpion spiking neuron model in an artificial environment. With this system we were able to localize a moving target with precision of 4.05° ± 3.01° under laboratory conditions and continuous single sine wave stimuli. Acquisition, pre-processing, transmission, analysis, and finally visual display of the results work reliably. Thus, the first step toward a device capable of locating sound sources is accomplished.

Although in these tests no precautions were taken during data collection to avoid interference with unintended noise sources such as colleagues talking, traffic, ACs, reflections, etc. This might indicate a robustness of the spiking neural network model against ambient noise during constant stimulation.

One limitation for localization is that the behavior of the recent model it is not predictable when having multiple non-continuous sound sources. When one source falls silent the model immediately switches to the source with highest amplitude and inhibits the opposite microphones. Thus, so far it works best with continuous stimuli, e.g., a continuous sine wave. One idea to overcome this is to estimate the direction of the source over a longer period to limit the impact of the silent period or to introduce an “afterglow effect” where the opposite microphones are not immediately released but remain inhibited for a predefined period. With this measure it is more likely that the model catches up on the initial sound source after a pause, e.g., a speaker.

In the next step, we want to bring the SNN model one step closer toward real-time processing by automatizing the data management and analysis. Additionally, the results indicate that, by varying different time parameters of the neural network, the performance of the SNN can be improved, and most probably reach the performance reported for the analog neural network, where the error is in the low single-digit range and the standard deviation at 4.5° (van Schaik and Shamma, 2003, 2004) or even the ASU units with CMOS VLSI design, which is reported to be around 1°–2° (Julian et al., 2006; Goodman and Brette, 2008; Chacon-Rodriguez et al., 2009). As the neural network is very simple it is expected that the performance parameters are advantageous, which we want to investigate and prove an implementation in custom hardware, using a CPLD/FPGA or even new memristive devices (Kyriakides et al., 2012) is feasible.

## Author contributions

CB: hardware part and setup. GG: SNN model and simulation. JG: idea, discussion, and supervision.

### Conflict of interest statement

The authors declare that the research was conducted in the absence of any commercial or financial relationships that could be construed as a potential conflict of interest.

## Footnotes

^1^Meetvideo video conference system.

^2^Polycom eagleeye director.

^3^Univideo live streaming solution.
